# *Candida* Infections and Their Prevention

**DOI:** 10.5402/2013/763628

**Published:** 2012-11-04

**Authors:** M. Anaul Kabir, Zulfiqar Ahmad

**Affiliations:** ^1^Molecular Genetics Laboratory, School of Biotechnology, National Institute of Technology Calicut, Calicut 673601, India; ^2^Department of Biological and Environmental Sciences, Alabama A&M University, Normal, AL 35762, USA

## Abstract

Infections caused by *Candida* species have been increased dramatically worldwide due to the increase in immunocompromised patients. For the prevention and cure of candidiasis, several strategies have been adopted at clinical level. *Candida* infected patients are commonly treated with a variety of antifungal drugs such as fluconazole, amphotericin B, nystatin, and flucytosine. Moreover, early detection and speciation of the fungal agents will play a crucial role for administering appropriate drugs for antifungal therapy. Many modern technologies like MALDI-TOF-MS, real-time PCR, and DNA microarray are being applied for accurate and fast detection of the strains. However, during prolonged use of these drugs, many fungal pathogens become resistant and antifungal therapy suffers. In this regard, combination of two or more antifungal drugs is thought to be an alternative to counter the rising drug resistance. Also, many inhibitors of efflux pumps have been designed and tested in different models to effectively treat candidiasis. However, most of the synthetic drugs have side effects and biomedicines like antibodies and polysaccharide-peptide conjugates could be better alternatives and safe options to prevent and cure the diseases. Furthermore, availability of genome sequences of *Candida* *albicans* and other non-*albicans* strains has made it feasible to analyze the genes for their roles in adherence, penetration, and establishment of diseases. Understanding the biology of *Candida* species by applying different modern and advanced technology will definitely help us in preventing and curing the diseases caused by fungal pathogens.

## 1. Introduction

*Candida* species are associated with human beings for quite long time as harmless commensals. They are commonly found on the mucosal surfaces of gastrointestinal and genitourinary tracts and skin of humans. However, they become opportunistic pathogens in immunologically weak and immunocompromised patients. As opportunistic pathogens, they can cause local mucosal infections and sometimes, systemic infections in which *Candida* species can spread to all major organs and colonize in these organs [1, 2]. The systemic infections can be life threatening among the individuals having severely paralyzed immune system such as AIDS patients, people undergoing chemotherapy and radiotherapy treatment for cancers, and patients undergoing organ transplants. As the number of immunocompromised patients is increasing worldwide due to change in life style and improvement in medical facilities, infections caused by *Candida* species and mainly by *Candida albicans* have been increased dramatically in the last two decades. This has posed a serious and daunting challenge to the effective management of candidiasis and cost has been increased manifold. It is estimated that in the United States itself the excess cost due to candidemia is between $1 and $2 billion per year [3, 4]. Here we briefly review different aspects of *Candida* infections, antifungals for treatment of candidiasis, drug resistance, and certain preventive measures. 

## 2. *Candida* Infections

*Candida* species can cause superficial and local mucosal infections and the best known of these is commonly called thrush. Such infections generally affect gastrointestinal, vaginal, esophageal, and oralpharygeal mucosae. Besides, most of the women suffer from vulvovaginal Candidiasis (VVC) at least once in their life time [5]. Some women experience repeated recurrences of this infection and it is known as recurrent vulvovaginal candidiasis (RVVC). The oral-pharyngeal candidiasis (OPC) is common among the HIV-infected patients and it is considered as an important marker for the onset of AIDS as well. OPC also affects oral cancer patients and debilitated patients who produce less amount of saliva [6]. However, it can cause a severe, life-threatening bloodstream infection that leads to colonization of *Candida* in internal organs (disseminated candidemia) which poses serious health problem in these individuals. Mortality rate for these patients is observed between 30% and 50% [7, 8]. *Candida* infections in the United States are the fourth most common hospital acquired infections and the second most common cause due to such infections [7]. Among the *Candida* species, *C. albicans* causes most of the candidemia, followed by non-*albicans* strains such as *Candida glabrata*, *Candida tropicalis*, *Candida parapsilosis,* and *Candida krusei* [9]. *C. glabrata* is responsible for about 16% of all bloodstream infections whereas *C. krusei* accounts for 2% of all the clinical *Candida* isolates [10, 11].

## 3. Antifungal Drugs and Mechanism of Action

For the effective treatment of superficial mucosal infections and systemic life-threatening fungal diseases, a considerably large number of antifungal drugs have been developed and used for clinical purposes (Table 1). Though fungal infections were known for centuries, antifungal drugs were not available till 1930s. The first antifungal drug griseofulvin was isolated as a metabolic product from the mold *Penicillium griseofulvum* in 1939. However, it took several years to prove its efficacy in curing fungal infections and it was not used for clinical purposes till 1958 [12]. Subsequently, antifungal drug in the category of polyene, amphotericin B, was introduced for clinical purpose in 1960 which was much more effective and even today it is considered as one of the best antifungals [13]. However, to counter the growing challenges of fungal infections and increasing demands of appropriate drugs, many potential antifungal drugs have been developed since 1960s onward and are being used to treat fungal infections. Here we will give brief descriptions of some of these drugs. 

### 3.1. Azole Antifungal Drugs

 Azole drugs are one of the most common classes of drugs used for treatment of *Candida* infections worldwide for both mucosal and systemic infections. The azole derivatives were introduced as antifungals in 1960s and it is most rapidly expanding (Table 1) [30–32]. Azole drugs are categorized as imidazole or triazoles depending upon the presence of two or three nitrogens in the five-membered azole ring. Most of the azole derivatives are fungistatic having broad spectrum against yeast and filamentous fungi. These antifungals target ergosterol biosynthetic pathway and thereby inhibit the growth of fungi [14–17]. Ergosterol is the major component of fungal cell wall and acts as a bioregulator for maintaining fluidity and asymmetry of cell membrane and overall integrity of the cell wall [33–35]. Azole drugs such as fluconazole, itraconazole, voriconazole, and posaconazole inhibit the lanosterol 14*α*-demethylase encoded by the gene *ERG11* and decrease the level of ergosterol required for cell membrane [18–20]. On the other hand, the precursors of ergosterol, such as lanosterol, 4,14-dimethly zymosterol, and 24-methylene dihydrolanosterol, are accumulated inside the cell and integrated into plasma membrane resulting in the altered structure and function of the membrane. Subsequently, it increases water penetration and drug uptake into the cell [36, 37]. The azole-induced altered plasma membrane structure also leads to several other responses in the cell including inactivation of vacuolar ATPases (V-ATPase), inhibition of hyphal development, and change in the oxidative and nitrosative stresses [38–40].

### 3.2. Polyenes

The polyene antibiotics, produced by *Streptomyces* species, have broader spectrum than many other antifungal drugs and they are fungicidal in nature instead of fungistatic like azole drugs [41–43]. The most commonly used polyenes are amphotericin B, nystatin, and natamycin. These drugs act by binding specifically to ergosterol present in the plasma membrane and thereby affecting the integrity of cell membrane that results in cell death. Matsumori et al. have shown that amphotericin B has direct intermolecular interaction with ergosterol whereas it scarcely interacts with mammalian counterpart, cholesterol [44]. This intermolecular interaction has also been supported by other evidences such as higher affinity of amphotericin B to ergosterol-containing membranes than to sterol-free and cholesterol membranes [45, 46]. The complex formation between the polyenes and ergosterol causes disruption in the membrane by forming membrane-spanning ion channels that result in the increased permeability of the membrane, leakage of essential components, and death of the pathogens [21, 47]. Several studies have also suggested that polyenes can cause oxidative damages to the cell that contributes to their fungicidal activity [22, 41, 48]. Undoubtedly, amphotericin B has broad specificity against many fungal pathogens; however, it has considerably high toxic effect on human cells leading to renal failure in the patients undergoing this treatment. For reducing this toxicity but retaining the full activity of amphotericin B, new formulations, such as liposome, lipid complexes, and colloidal dispersions, have been made and obtained promising outcomes [49–55].

### 3.3. Echinocandins

 These compounds are a class of lipoproteins, discovered in the 1970s, having fungicidal activity against *Candida* both *in vivo* and *in vitro* [56–58]. The commonly used echinocandins, for clinical purposes are caspofungin, micafungin, and anidulafungin [23–26]. These drugs are specific noncompetitive inhibitors of the enzyme *β*-(1,3)-glucan synthase, a membrane heterodimeric protein, responsible for the synthesis of *β*-glucan [59]. A recent study has shown that anidulafungin, a semisynthetic echinocandin has better efficacy compared to commonly used fluconazole for systemic candidiasis caused by *C. albicans* [60]. This echinocandin has more effective global response compared to fluconazole and cleans the bloodstream infections quite faster. Moreover, after treatment with this drug, a fewer persistent infections have been observed [60]. This interesting outcome might be attributed to the fungicidal activity of echinocandin (anidulafungin) which could have better response in the patients compared to fungistatic fluconzaole. However, this observation cannot be extrapolated to other systemic infections and the patient's immunological status might contribute to efficacy of drugs used. 

### 3.4. Allylamines

 The most commonly used allylamines for clinical purposes include naftifine and terbinafine [27]. Allylamines are noncompetitive inhibitors of squalene epoxidase and are effective against many fungal agents including azole-resistant *Candida* strains [61]. The enzyme squalene epoxidase is encoded by the gene *ERG1* located early in the ergosterol biosynthetic pathway [62]. Cells treated by allylamines accumulate squalene while becoming deficient in ergosterol (essential component of cell membranes) as subsequent steps in the ergosterol biosynthetic pathway are blocked. Furthermore, studies with isolated squalene epoxidase indicated that this enzyme is indeed the target of allylamines [61]. The fungal cell death by allylamines may not be due to the depletion of ergosterol in the cell as such, rather it could be because of accumulation of squalene that results in the formation of altered plasma membrane and disruption of membrane organization. This leads to increased permeability of membrane resulting in the cell death [27, 63]. It has also been demonstrated that naftifine has anti-inflammatory properties such as reduction in polymorphonuclear leukocyte chemotaxis and reduction in superoxide production. Though naftinfine has shown good efficacy for fungal treatments and relief of inflammatory signs and symptoms, it has some adverse effects like burning or stinging at the site of application [64].

### 3.5. Fluorinated Pyrimidine Analog

**Figure 1:**
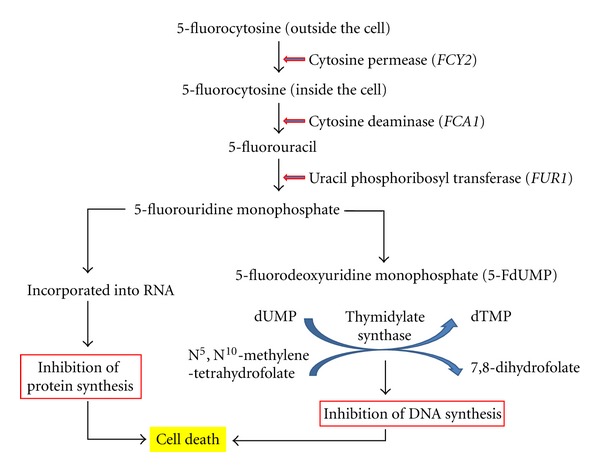
Schematic diagram of the effect of 5-Fluorocytosine (5-FC) on the fungal cell. Genes for three enzymes are given in italic capital letters in the bracket. Mutations in these genes make the cells resistant to 5-FC.

The 5-fluorocytosine (flucytosine or 5-FC) is a derivative of cytosine (essential component of nucleic acids) and was first synthesized in 1957 as anti-tumor drug [65]. However, its efficacy was not proven in cancer treatment. Later, it was tested for its antifungal activity, and subsequently, it was used for treatment of fungal infections in 1968 especially for candidiasis and cryptococcosis [66, 67]. Flucytosine does not have any antifungal activity as such, rather its metabolite 5-fluorouracil (5-FU) is considered to be toxic for the fungal cell. It may be asked why 5-FU is not administered to patients suffering from candidiasis when it has toxic activity rather than giving 5-FC. The reason is that 5-FU is toxic to mammalian cell, whereas 5-FC is quite safe. Here mode of action of 5-FC is discussed briefly. 5-FC is taken up by *Candida *species through cytosine permease and once inside the cell, it is rapidly converted into 5-FU [28, 29]. This 5-FU can exert its toxic effect by adopting two different pathways inside the cell. In one pathway, 5-FU is converted into 5-fluorodeoxyuridine monophosphate (FdUMP) which is proven to be potential inhibitor of thymidylate synthase, an essential enzyme for biosynthesis of thymidine [68, 69]. As a result, DNA synthesis gets blocked in fungal cells and it is unable to go for cell division. Another mechanism is through the conversion of 5-FU into 5-fluorouridine monophosphate and subsequently into 5-fluorouridine triphosphate which is incorporated into RNA in place of normal uridine triphosphate. In turn, this inhibits the protein synthesis in fungal cell (Figure 1) [68, 70, 71]. Therefore, both processes prove to be lethal for fungal pathogens and, subsequently, they are eliminated from the site of infection. 

## 4. Drug Resistance

**Figure 2:**
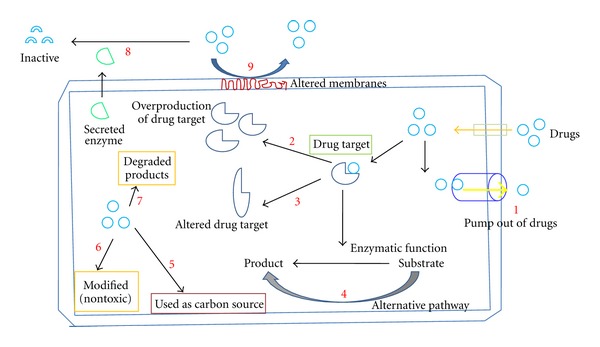
Probable mechanisms of drug resistance in *Candida* species. (1) Drugs are pumped out by efflux pump. (2) Drug targets such as enzymes are overproduced and drugs cannot inhibit the enzymatic reactions. (3) Due to mutations, the structures of enzymes or other proteins are altered and drugs cannot bind to it. (4) Crucial enzymatic function that is inhibited by drug can be bypassed. (5) Drugs may be degraded and are used as carbon source. (6) Drugs may be modified by enzymes and become nontoxic. (7) Drugs are degraded and become nonfunctional. (8) Extracelluar enzyme may degrade the drugs outside the fungal cell and make them inactive. (9) Altered membrane may inhibit the entry of drugs into cell and drugs cannot function.

Though infections caused by *Candida* species are treated with different antifungal drugs available as mentioned above, the drug resistance is posing a serious problem to the health of individual patients and management of health care system becomes difficult. Studies have shown that several factors including pumping out of drugs from fungal cells, modification of the targets by incorporating point mutations in the genes, modification of key enzymes for biosynthetic pathways, and modulation of transcription factors play important roles for this phenomenon (Figure 2) [72, 73]. These mechanisms are discussed below briefly. 

### 4.1. Efflux Pumps

 Efflux pumps remain the major reason for drug resistance in almost all the *Candida* species as they have broad specificity and thought to be a prominent factor for drug resistance in clinical isolates. There are two major classes of efflux pumps, ABC (ATP binding cassette) transporter and MFS (major facilitator superfamily) pump. These transmembrane proteins transport different substrates across membranes using two different energy sources. The ABC transporters use ATP as energy source whereas MFS pump utilizes proton-motive force across the membrane. Among the ABC transporters, Cdr1p and Cdr2p have been well studied and they play an active and critical role in drug resistance in *C. albicans* [74–77]. Also, the role of Mdr1p (member of MFS pump) has been demonstrated in drug resistance in *Candida* species. Several studies have shown that azole-resistance of clinical isolates of *Candida* species is always associated with the overexpression of Cdr1p and Cdr2p as well as Mdr1p. In addition to azole drugs, Cdr1p and Cdr2p are also implicated in the drug resistance to topical antifungals such as terbinafine and amorrolfine [78]. Now the question is how do these pumps efflux drugs from fungal cells? Structure-function analysis of Cdr1p and Cdr2p shows that these ABC transporters have two distinct domains, the transmembrane domains (TMDs) and the nucleotide binding domains (NBDs). It is suggested that two TMDs in the homodimer generate inward-facing drug binding cavity in which drugs can bind either from lipid bilayer or from cytoplasm. Subsequently, binding of two ATP molecules to two NBDs induces conformational changes in TMDs resulting in the opening of drug binding cavities extracellularly and closing intracellularly. This allows the bound drugs to be effluxed from the cell. Again, the hydrolysis of bound ATP resets this pump in the drug-binding mode. Thus, it completes one cycle and this is repeated to efflux drugs from fungal cell making it resistant to that particular drug. In the absence of crystal structure of ABC transporter, the above mechanism has been deduced from ABC transporter Sav1866 of *Staphylococcus aureus* whose crystal structure is available along with AMP-PNP [73, 79–81]. 

### 4.2. Mutations in the Target Sites

 Mutations have been observed in a number of genes in clinical strains of *Candida* species which are resistant to antifungal drugs. For becoming resistant to a particular drug, a specific mutation in a specific gene has to occur. For example, mutations in the gene, *ERG11*, encoding sterol 14*α*-demethylase can reduce the binding of azole drugs to this enzyme resulting in the increased resistance of the *Candida* strains to these drugs [82, 83]. Cross-resistance to different azole drugs has also been observed in the strains having mutations in *ERG11* [84]. Mutations that affect the uptake of 5-FC or its conversion into 5-FU and incorporation into growing nucleic acid chains have been implicated in the drug resistance as well [85, 86]. For example, most of the 5-FC-resistant *Candida* strains have mutations in *FUR1* gene that encodes uracil phosphoribosyl transferase and the mutant version of this enzyme prevents the conversion of 5-FU to FdUMP. Studies have shown that mutation in *FUR1* occurs at 301 bp position of the gene resulting in amino acid change from arginine to cysteine at 101 position in Fur1p [87]. Also the mutations, glycine to aspartate at position 28 and serine to leucine at position 29 in the enzyme cytosine deaminase, encoded by the gene *FCA1*, have been implicated in the resistance to 5-FC for *C. albicans* [88]. Furthermore, in a recent study, mechanism of resistance to 5-FC with respect to mutations in *FCA1* has been analyzed extensively in *C. glabrata* [89]. Mutations are also found in the gene *FCA1* (also known as *FCY1*) of clinical isolates of *C. dubliniensis* and *C. lusitaniae *which are resistant to 5-FC [85, 90]. Similarly, mutations in the gene *FKS1* that encodes a subunit of *β*-1,3-glucan synthase complex can cause resistance to echinocandin drugs as well [91]. 

## 5. Diagnosis and Prevention of Candidiasis

 Prevention of candidiasis and its management broadly depends on two important and critical factors. One is the early detection and identification of *Candida* strains. Second is the use of appropriate antifungal drugs. For example, *C. albicans* is quite sensitive to azole drugs whereas non-*albicans* strains such as *C. glabrata* and *C. krusei *are resistant to this antifungal. Here we will give brief account of these two factors. 

### 5.1. Identification of *Candida* Species

 The early detection of the strains certainly facilitates the use of antifungal drugs in cost-effective manner and will have positive impact on overall health of the patients [92]. The techniques for identification of *Candida* species must be rapid and strain-specific that not only separate *Candida* from other microbial pathogens rather it should also be able to distinguish them from other important fungal pathogens such as *Aspergillus fumigatus*, *Cryptococcus neoformans,* and other yeasts. For the identification of *Candida* species, different methods have been developed and used since 1950s; however, most of them were based on culturing the strains and searching for different phenotypes which is considered to be time-consuming [93–96]. As a result, treatment of candidiasis faced major problems and mortality and morbidity rates were quite high. However, different advanced technologies have now been developed and are being used for rapid and accurate identification of *Candida* strains that help in early diagnosis, treatment and management of candidemia and other infections caused by *Candida* species. Here, three of the advanced techniques for *Candida* identification have been discussed briefly. 

#### 5.1.1. Polymerase Chain-Based *Candida* Detection

 A large number of different kinds of protocols have been developed over the last five decades to identify different fungal strains present in clinical specimens. The detection and speciation techniques include germ tube test, chromogenic test, enzymatic test, immunological test (identification of antigen or antibody), and fermentation tests [97, 98]. However, in many cases, these tests were not sensitive enough to give the accurate result, and subsequently, it delayed the antifungal therapy. Furthermore, blood culture test is considered to be “gold standard” for identification of *Candida* species, but it takes 24–48 hours to give the positive signal. Moreover, this method may not be sensitive enough to identify the strains from different tissue specimens due to low number of cells present in different internal organs especially in case of invasive candidiasis. However, this problem has been addressed effectively by applying polymerase chain reaction (PCR) for identifying *Candida* species. Recent years have witnessed the development of very sensitive PCR machines that are able to detect and separate large number of fungal pathogens including *A. fumigatus*, *C. neoformans,* and *C. albicans*. Different *Candida* DNA markers such as 5.8S rRNA genes, 18S rRNA gene, small subunit rRNA gene, noncoding internal transcribed spacer (ITS) of rRNA genes, and lanosterol demethylase gene have been used in PCR amplification for detection of *Candida* species [99–105]. This technique has been improved further to separate *C. albicans* from other *Candida* by PCR amplification of 5.8S rRNA gene followed by DNA enzyme immunoassay with *C. albicans*-specific oligonucleotide probe [106]. Later on, real-time PCRs were developed for rapid and accurate identification of different *Candida* species which is more sensitive and less time-consuming [107–110]. Real-time PCRs have been improved further to identify different *Candida* species within reasonably short time [111–113]. For example, Metawally et al. adopted real-time PCR in which rRNA gene complex has been used as target sequence for amplification that differentiates between fluconazole-sensitive and resistant *Candida* strains. This technique is able to identify the most commonly encountered *Candida* species in blood cultures such as *C. albicans, Candida parapsilosis, Candida tropicalis, C. dubliniensis, C. glabrata, *and* C. krusei* in less than 3 hours [111]. On the other hand, Innings et al. have been able to identify eight *Candida* species such as *C. albicans, C. tropicalis*, *C. parapsilosis, C. glabrata, C. famata*, *C. dubliniensis,* and *C. guilliermondii,* commonly found in blood culture, by amplifying RNaseP RNA gene *RPR1* sequence [112]. Recently it has been shown that there is no significant difference between SeptiFast (a commercially available molecular diagnosis of sepsis based on PCR) and blood culture method in the identification of pathogens in sepsis patients. However, the combination of both methods might be quite helpful for patients with suspected sepsis and especially those who are undergoing antibiotic treatment in an internal medicine ward in hospital [114]. In another study, two commercially available universal rRNA gene PCR plus sequencing test, SepsiTest and universal microbe detection (UMD), were evaluated for suspected infectious endocarditis (IE). These tests proved to be of immense value for rapid diagnosis of IE, particularly for cases of culture-negative infections [115].

#### 5.1.2. MALDI-TOF-MS for *Candida* Detection

 Matrix-assisted laser desorption ionization time-of-flight mass spectrometry (MALDI-TOF-MS) was introduced by Karas and Hillenkamp in the late 1980s for mass determination of proteins [116]. This technique proved to be extremely powerful for the analysis and identification of other large biomolecules such as nucleic acids, carbohydrates, and lipids [117–119]. This has been extensively used to profile, characterize, and identify proteins and other molecules from intact and disrupted cells. Subsequently, the power of this spectrometry has been exploited for the rapid identification of clinically important bacteria and yeasts [120–122]. In the recent years, this technology has been applied to *Candida* biology as well as for rapid, accurate, and cost-saving identification of different *Candida* species and also for their speciation [123–127]. For example, MALDI-TOF intact cell mass spectrometry (MALDI-TOF-ICMS) has been extremely useful for separating *Candida* species that are not easy to differentiate in the conventional phenotypic growth or biochemical reactions. This technology has been applied to separate *Candida* species such as *C. parapsilosis*, *C. orthopsilosis*, and *C. metapsilosis *as well as closely related species like *C. dubliniensis*, *C. albicans* and *C. glabrata* in a time-saving manner [125]. In another study, Sendid et al. have compared the suitability of MALDI-TOF-MS for the identification of *Candida *species with that of conventional identification (CI) methods such as morphological, biochemical, or immunological procedures. Concordance between MALDI-TOF-MS and CI was found to be 98–100% for medically important pathogens and was able to separate closely related *Candida* species such as *C. albicans, C. dubliniensis*, and other *Candida* strains [127]. Taken together, these studies have clearly shown the potential of MALDI-TOF-MS for rapid, accurate and cost-saving identification of *Candida* species that will lead to appropriate antifungal therapy in a timely manner. 

#### 5.1.3. DNA Microarray for *Candida* Detection

DNA microarray has revolutionized the understanding of molecular functioning of different genes in all the organisms including humans. Though it was invented with respect to the analysis of gene expression, it is now applied to understand different aspects of molecular biology including rapid detection and identification of different viruses, bacteria, and fungi of medical importance for proper diagnosis and therapy [128–130]. In brief, probes (oligonucleotides, short fragment of DNA or cDNA) can be spotted on solid matrix (glass slides, plastic, or biochip) and the targets are amplified from mRNA or genomic DNA and labeled with different fluorophores. Fluorescently labeled target sequences are hybridized with probes and the signals generated from interaction between targets and probes are analyzed. The strength of the signal from a particular spot of array will depend on the amount of target sequence present in that spot binding to the probe. 

In the oligonucleotide microarray method, specific probes targeted to internal transcribed spacer 2 (ITS2) can be used for hybridization with fungal DNA amplified by PCR from different species. This technique is sensitive enough to discriminate among different fungal pathogens at species level and can detect as minimum as 15 pg of DNA/mL in the sample [131]. This method has been further improved by enhancing the hybridization signals with gold nanoparticles and silver deposition and detection using flatbed scanner [132]. This advanced method has been very sensitive and can detect *C. albicns* in the sample as low as 10 cells/mL. For the rapid identification of microbes in bloodstream infections (BSI), DNA-microarray-based Prove-it Sepsis assay has been evaluated and found to be 98-99% sensitive compared to conventional blood-culture tests. It takes less than 3 hours from DNA extraction to BSI diagnosis [130]. Undoubtedly, DNA microarray with its different variants will be quite helpful for rapid and accurate detection and identification of different fungal pathogens including *Candida* species. This will certainly complement other available methods for proper diagnosis of fungal infections.

### 5.2. Prevention and Treatment of Candidiasis with Biomedicine

 Though the number of antifungal drugs is rapidly increasing and they are used to treat *Candida* infections for both mucosal and invasive, the outcome is not satisfactory so far. Moreover, most of the antifungal drugs have substantial amount of toxic effect on human cells. Therefore, it has been imperative to find an alternative to the conventional drugs to treat the infected patients. Besides, it will be better to prevent the onset of the diseases instead of curing it. This can be done by adopting certain immunization strategies as it is done for many other bacterial infections [133–136]. Though the concept of protection through antibody has been controversial for quite long time, a large amount of data is coming out in favor of its use to prevent and also to cure the diseases. This alternative method is gaining its importance in the context of growing number of immunocompromised patients who are sensitive to toxic effect of conventional drugs. For treating the *Candida* infections, antibodies have been generated against cell wall polysaccharides, heat shock protein, secreted proteins, and peptides [137–141]. The synthetic glycopeptide vaccine against disseminated candidiasis has been found to be quite effective in mice [140]. Furthermore, synthetic glycopeptide conjugates were made by combining fungal cell wall beta-mannan trisaccharide and six 14 mer peptides from six different proteins such as enolase, phosphoglycerate kinase, fructose-bis-phosphate aldolase, hyphal wall protein-1, methyl tetrahydropteroyltriglutamate, and glyceraldehydes-3-phosphate dehydrogenase [140]. Furthermore, it has been demonstrated that vaccine and monoclonal antibody E2-9 (IgM) against peptide, Fba (derived from fructose bis phosphate aldolase), can protect mice from candidiasis [142]. Also, antibodies raised against beta glucan (elicited by peptide conjugate) are able to protect mice that are challenged with *C. albicans* possibly by inhibiting the fungal growth and its adherence to mammalian cell [143, 144]. Among the antibodies that are used for prevention as well as for curing of *Candida* infections, Mycograb (human recombinant antibody generated against Hsp90) has been of utmost importance in the last one decade. This antibody has been used in combination with other antifungal drugs and produced quite encouraging result [145, 146]. Matthews et al. have reported that Mycograb is active against a range of *Candida* species such as *C. albicans, C. krusei, and C. tropicalis *and it has synergistic effect on amphotericin B [139]. In another study, Mycograb was used in combination with lipid-associated formulation of amphotericin B for the treatment of invasive candidiasis and shows promising result [145]. However, recently, it has been shown that potentiation of amphotericin B appears to be nonspecific protein effect rather than the effect of antibody [146]. Efungumab (monoclonal antibody against Hsp90) has been tested in combination with other antifungal drugs for treatment of *Candida* infections and also for prevention [147–150]. Furthermore, as the complete genome sequences of quite good number of *Candida* species including *C. albicans, and C. glabrata, C. dubliniensis *are available, it is possible to develop genetically engineered *Candida* strains which are avirulent and can be used for immunization as vaccines. Also, *Candida*-specific genes or their protein products can be used as biomedicine to prevent candidiasis. Taken together, it seems plausible to take an alternative method for vaccination and prevention of *Candida* infections.

## 6. Conclusion

 It is well accepted that *Candida* infections are on the rise and it needs to be handled with due care to decrease the rate of morbidity and mortality for immunocompromised patients. For the proper management of the *Candida* infections, multiple strategies must be adopted in a cost-effective and time-saving manner. First strategy will be to prevent the onset of disease by immunization/vaccination of the susceptible individuals by applying knowledge gained from genomics, proteomics, and transcriptomics of *Candia* and related species. Second strategy is to treat the *Candida* infections seriously and promptly. Any delay for antifungal therapy may lead to disseminated candidemia and systemic candidiasis in which different internal organs will be highly colonized by *Candida* strains. Again for proper antifungal therapy, it is imperative to identify the *Candida* species at early stage of infections. The conventional methods such as phenotypic, morphological, biochemical, and immunological should be replaced with highly advanced technologies like MALDI-TOF-MS and real-time PCR and DNA microarray in clinical setup. Identification and speciation facilities should be developed in such a way so that whole process will be rapid, accurate, cost-effective, and time-saving. 

Once the strains are identified, appropriate antifungal drugs can be administered to the patients and level of fungal strains can be monitored in clinical specimens. However, almost all the *Candida* strains isolated from infected individuals are becoming resistant to the commonly used antifungal drugs. In this regard, combination of two or more drugs has been suggested and tested for *C. albicans* and other *Candida* species and found to be synergistic for amphotericin B/ketoconazole, 5-FC/ketoconazole, and other combinations as well [151, 152]. The drug combination therapy was also tested in mice model and patients [153, 154]. In a recent study, Tavanti et al. have shown that clinical isolates of *C. glabrata* (low susceptibility to azole drugs) are susceptible to human cationic peptide hepcidin (Hep-20) (100–200 *μ*g/mL). However, increased antifungal activity was observed when combined with amphotericin B and a synergistic effect was found for Hep20/caspofungin and He-20/fluconazole combinations [155]. 

Another measure to counter the rising drug resistance of the strains is to use inhibitors for efflux pumps in combination with commonly used drugs. The inhibitors for ABC pumps such as milbemycins, enniatin, FK506, FK520, and unnarmicins can be used along with azole drugs to reverse the drug resistance [73, 156–159]. Recently, Hayama et al. have assessed the therapeutic potential of D-octapeptide derivative RC21v3 (an inhibitor of Cdr1p) in a murine oral candidiasis infection model and have shown its potential in combination with fluconazole [160]. This suggests that this inhibitor has a potential in treating oral candidiasis. In another study, Holmes et al. have reported the identification of the monoamine oxidase A inhibitor, clorgyline, as inhibitor of ABC and MFS pumps in clinical isolates of *C. albicans* and *C. glabrata* [161]. 

However, for the prevention of onset of the disease and to treat the *Candida* infections effectively, the understanding of the complete life cycle of *C. albicans* and other *Candida* species is required. In this regard, the functions of all the ORFs and specially the *Candida*-specific genes/ORFs should be assigned. This will help in developing potential antifungal drugs in terms of antibody, proteins, DNA, or the whole-organism itself for the prevention of this disease. 

## Figures and Tables

**Table 1:** Commonly used antifungal drugs and their targets/mode of action.

Antifungals	Targets/mode of action	References
Azoles	Ergosterol biosynthesis (inhibition of *ERG11* gene product, lanosterol 14*α*-demethylase)	[14–20]
Fluconazole (FLC)		
Itraconazole (ITC)		
Voriconazole (VCZ)		
Posaconazole (POS)		
Ravuconazole		
Isavuconazole		
Pramiconazole		
Albaconazole		
Miconazole		
Ketoconazole		
Polyenes	Cell membrane ergosterol (increased permeability and oxidative damage)	[21, 22]
Amphotericin B		
Nystatin		
Echinocandins	Cell wall biosynthesis, inhibition of *GSC*1 gene product, *β*(1,3)-glucan synthase	[23–26]
Caspofungin		
Micafungin		
Anidulafungin		
Allylamines	Ergosterol biosynthesis (inhibition of *ERG1* gene product squalene epoxidase)	[27]
Terbinafine		
Naftifine		
Fluorinated pyrimidine	DNA and RNA synthesis (misincorporation of 5-fluorouracil)	[28, 29]
analogs		
5-fluorocytosine		
